# Carbonic Anhydrase IX (CAIX), Cancer, and Radiation Responsiveness

**DOI:** 10.3390/metabo8010013

**Published:** 2018-02-10

**Authors:** Carol Ward, James Meehan, Mark Gray, Ian H. Kunkler, Simon P. Langdon, David J. Argyle

**Affiliations:** 1 Cancer Research UK Edinburgh Centre and Division of Pathology Laboratory, Institute of Genetics and Molecular Medicine, University of Edinburgh, Edinburgh EH4 2XU, UK; j.meehan@hw.ac.uk (J.M.); s9900757@sms.ed.ac.uk (M.G.); I.Kunkler@ed.ac.uk (I.H.K.); simon.langdon@ed.ac.uk (S.P.L.); 2 Royal (Dick) School of Veterinary Studies and Roslin Institute, The University of Edinburgh, Easter Bush, Midlothian EH25 9RG, UK; david.argyle@roslin.ed.ac.uk; 3 Institute of Sensors, Signals and Systems, School of Engineering and Physical Sciences, Heriot-Watt University, Edinburgh EH14 4AS, UK

**Keywords:** carbonic anhydrase IX, cancer, hypoxia, radiation, resistance

## Abstract

Carbonic anhydrase IX has been under intensive investigation as a therapeutic target in cancer. Studies demonstrate that this enzyme has a key role in pH regulation in cancer cells, allowing these cells to adapt to the adverse conditions of the tumour microenviroment. Novel CAIX inhibitors have shown efficacy in both *in vitro* and *in vivo* pre-clinical cancer models, adversely affecting cell viability, tumour formation, migration, invasion, and metastatic growth when used alone. In co-treatments, CAIX inhibitors may enhance the effects of anti-angiogenic drugs or chemotherapy agents. Research suggests that these inhibitors may also increase the response of tumours to radiotherapy. Although many of the anti-tumour effects of CAIX inhibition may be dependent on its role in pH regulation, recent work has shown that CAIX interacts with several of the signalling pathways involved in the cellular response to radiation, suggesting that pH-independent mechanisms may also be an important basis of its role in tumour progression. Here, we discuss these pH-independent interactions in the context of the ability of CAIX to modulate the responsiveness of cancer to radiation.

## 1. Introduction

During growth, many solid tumours develop areas of low oxygen tension, or hypoxia, caused by malformation of the tumour vasculature and the increasing distance of tumour cells from the capillary bed. In tissues, O_2_ concentrations of 2–9% are typical, O_2_ concentrations ≤2% O_2_ are defined as hypoxic, and ≤0.02% are defined as severely hypoxic [1]. The diffusion distance of O_2_ from capillaries is approximately 100–200 μm; tumour cells situated further than this become hypoxic, as oxygen gradients develop in the tumour [2,3]. Circulation in the tumour is often cyclic, causing periods of acute or chronic hypoxia [4]. Tumour pH also falls with increased distance from blood vessels, with decreases from 7.4 to 6.0 measured around 300 μm from the vasculature [5]. However, the intracellular pH (pH_i_) of tumour cells is maintained between 7.0 and 7.4, by the actions of pH regulating proteins [6]. Poor perfusion also inhibits the removal of waste metabolites from the tumour, allowing acidosis to develop within the tumour microenvironment (TME). Hypoxia and acidosis cause major problems in cancer treatment, contributing to increased levels of resistance to both radiotherapy and chemotherapy [7].

## 2. Survival Strategies

To survive in these adverse conditions, cancer cells must adapt or die [8]. As one of the mechanisms of adaptation, cells in hypoxic conditions activate the transcription factor hypoxia inducible factor-1 (HIF-1), consisting of a heterodimer constructed from α and β subunits. In normal cellular O_2_ concentrations, the α subunit is rapidly degraded. The oxygen-dependent activation of prolyl hydroxylases causes hydroxylation of two proline residues (402 and 564) on HIF-1α, allowing interaction with an E3 ubiquitin ligase, VHL (the Von Hippel-Lindau factor), which targets this subunit for destruction in the proteasome [9]. This does not occur in hypoxic conditions and instead HIF-1α is stabilised and interacts with the HIF-1β subunit forming HIF-1, which activates gene transcription after nuclear translocation. Hypoxia-independent mechanisms for HIF-1α stabilisation additionally occur [10,11,12]. Other proteins also regulate HIF-1 activation, for example, factor inhibiting hypoxia inducible factor-1 (FIH-1), which prevents full activation of HIF-1 in moderate hypoxia. This protein maintains its activity in low O_2_ concentrations [13,14] by impairing the interaction between the C-terminal transactivation domain of HIF-1α and its co-activator proteins, causing partial activation of HIF-1 [15]. FIH-1 itself is inhibited in severe hypoxia, or by membrane type-1 matrix metalloproteinase/(MMP14), allowing full HIF-1 activation [13,16].

HIF-1 regulates the expression of genes involved in glycolysis, angiogenesis, pH regulation, migration, epithelial-mesenchymal transition (EMT), and invasion [17,18,19], many of which aid cancer progression. For example, EMT involves E-cadherin loss, which allows cancer cells to disperse and develop a migratory and invasive phenotype, and is also linked to increased resistance to chemotherapy and radiotherapy [20]. Hypoxia via HIF-1 causes E-cadherin loss by stimulating the lysyl oxidase (LOX)-Snail pathway [21]. LOX inhibition decreases the motility and invasiveness of cancer cells in hypoxia and also reduces metastasis *in vivo* [22]. Hypoxia also interferes with the homologous recombination, non-homologous end-joining, and mismatch repair DNA pathways, and inhibits the G1/S cell cycle checkpoint. This increases DNA errors and causes chromosomal instability [6,21].

**Figure 1:**
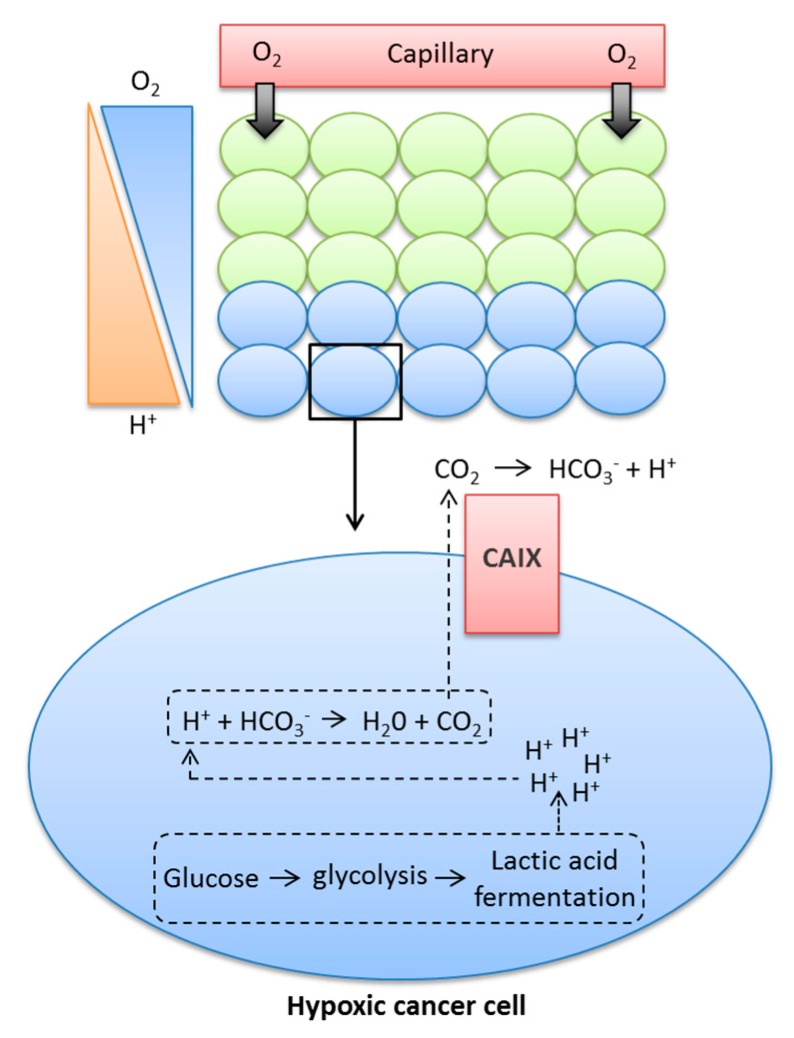
Contribution of CAIX to the movement of glycolytic protons from inside the cytoplasm to the extracellular milieu.

Cancer cells use aerobic glycolysis for energy and to provide components for cell growth and proliferation, even in normoxic conditions, causing higher rates of glycolysis and increased production of CO_2_, H^+^, and lactate [8,23]. These metabolic by-products must be removed from the cell to prevent the pH_i_ becoming acidic, and thus maintain a slightly alkaline pH_i_ consistent with survival. Early studies using D_2_O in yeast demonstrated that active transport mechanisms are likely to be dependent on protons, since deuterons could not substitute for protons in these processes [24], and further illustrated the role of alkaline pH_i_ in transformation, tumorigenicity, and proliferation [25,26]. Tumour cells can maintain their pH_i_ through increased expression and activation of pH regulatory proteins, some of which are HIF1-dependent, such as monocarboxylate transporter 4 (MCT4), which exports lactate and H^+^ from tumour cells, or carbonic anhydrase IX (CAIX), an enzyme that accelerates the conversion of CO_2_ and H_2_O to HCO_3_^−^ and H^+^ [18,27,28,29]. HCO_3_^−^ is transported back into tumour cells via HCO_3_^−^ transporters and used to buffer pH_i_ [28,30]. The role of CAIX is shown in Figure 1.

As a result of low O_2_ levels, hypoxic cancer cells are required to undergo lactic acid fermentation for the production of energy, a process that leads to the production of H^+^ ions. If these H^+^ ions are allowed to build up in the cytoplasm, they can lead to changes in pH, which can be detrimental to the cell. The metabolic acids generated within the cell can react with HCO_3_^−^, leading to the production of H_2_O and CO_2_. Membrane-permeant CO_2_ is a form in which much acid is removed from cancer cells. CAIX facilitates CO_2_ diffusion out of the cell by catalysing the extracellular hydration of CO_2_, leading to the production of H^+^ and HCO_3_^−^. CAIX therefore maintains a steeper efflux gradient for CO_2_, leading to a more alkaline intracellular pH, while also causing the acidification of the extracellular milieu.

**Figure 2:**
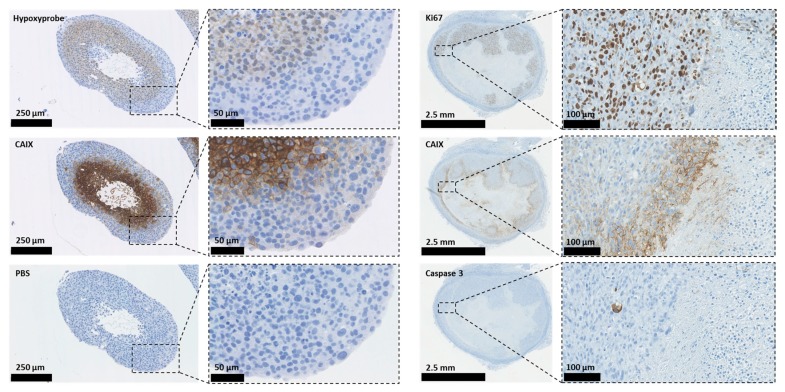
The identification of hypoxic areas and the expression of CAIX, proliferation, and apoptotic markers in 3D human breast cancer models.

Carbonic anhydrases (CAs) are ubiquitous metalloenzymes that catalyse the reversible formation of HCO_3_^−^ and H^+^ ions from H_2_O and CO_2_ [31]. At least 16 different isoforms of CAs have been isolated from mammals and differ in terms of cellular location, activity, and tissue locations. One CA, CAVI, is secreted, two (CAVA and VB) are found in the mitochondria, five are cytosolic (CAs I, II, III, VII, and XIII), and five are found on membranes (CA IV, IX, XII, XIV, and XV); of these, CAIX and CAXII have been shown to play an important role in cancer progression [32,33]. Some CAs have been shown to operate as part of transport ‘metabolons’ to increase the effectiveness of HCO_3_^−^- and H^+^-transporters [34,35,36,37]. This contributes to the maintenance of an alkaline pH_i_ in tumour cells and an acidic pH_e_ in the TME, which supports tumour growth, invasion, metastasis, and resistance to both chemotherapy and radiotherapy [5,6,38,39,40]. For example, in tumours, the most invasive regions are those exhibiting the lowest pH, which causes activation and increases expression of proteinases and metalloproteases that degrade components of the extracellular matrix (ECM), facilitating invasion and migration [5,41,42,43]. Alkaline pH_i_ causes resistance to apoptotic stimuli because caspase activation occurs in acidic pH_i_ conditions [44]; it also increases both DNA synthesis and cell proliferation, allowing tumour growth and progression [6,8,45,46]. Figure 2 illustrates the expression of CAIX, proliferation, the hypoxic region, and apoptotic staining in human 3D breast cancer models.

In Figure 2, the illustrations on the left demonstrate overlapping staining for CAIX and the hypoxic marker hypoxyprobe-1 in 3D spheroid cultures of HBL-100 human breast cancer cells. The right-hand illustrations demonstrate staining for CAIX, Ki67 (a proliferation marker), and caspase-3 (apoptosis) in a xenograft model of MDA-MB-231 human breast cancer cells.

Multiple studies have demonstrated a role for CAIX in pH regulation in cancer cells [28,47,48,49,50]. Targeting CAIX is proposed as a logical strategy for anti-cancer therapy, since it is an extracellular target, mainly associated with malignant growth, and is largely absent from most healthy tissue, with the exception of the gastro-intestinal tract and stomach [32,38,47,51,52]. CAIX staining in tumours is associated with poor prognosis and progression in several types of cancer, and in a series of lymph node-positive breast tumours it was found to correlate with metastasis [40,53,54,55,56,57,58]. Knockdown of CAIX in murine models leads to few phenotypic abnormalities other than gastric hyperplasia, inferring limited toxicity issues in normal tissue [59,60].

## 3. CAIX Inhibition as a Cancer Therapy

The effectiveness of CAIX inhibition as an anti-cancer strategy has been demonstrated in many pre-clinical studies using various cancer models. CAIX inhibitors negatively affect cancer cell viability and migration, as well as collagen destruction and invasion, and hinder both tumour formation and metastic growth in murine models, suggesting that increased expression and activity of CAIX in a cancer will adversly affect progression and prognosis. Many groups have now validated a crucial role for CAIX in growth, migration, invasion, and metastasis of tumours [40,52,61,62,63,64,65,66,67,68].

*In vitro* investigations have demonstrated that inhibition of CAIX using siRNA or CAIX inhibitors decreased the invasiveness of renal and ovarian cancer cells, while also reducing the amount of cells invading from human breast carcinoma spheroids [40,66,69,70]. A novel class of sulfamate CAIX inhibitors reduced the invasion, proliferation, and migration of human breast cancer cells, and also exhibited the capacity to reverse established invasion in a model consisting of breast tumour tissue from naïve biopsies [40,63,66,71]. CAIX knockdown significantly reduced the proliferation and survival of cancer cells under both normoxic and hypoxic conditions [40,66,72]. 

*In vivo* studies have shown that knockdown of CAIX can decrease tumour volume in both breast and colon cancer xenografts. Additive results could be obtained in co-treatments alongside antiangiogenic therapy. Knockdown also inhibited lung metastasis in breast cancer models [52,63,64]. CAIX overexpression studies in a colon cancer model demonstrated increased rates of tumour growth and expression of Ki-67, a marker of proliferation [64]. CAIX inhibitors also slowed tumour growth in breast cancer xenografts by decreasing proliferation and increasing cell death [40]. One of these inhibitors also exhibited anti-metastatic effects in a xenograft model of human breast cancer [73]. Interestingly, the use of CAIX inhibitors in *in vivo* systems did not lead to any reports of non-specific toxicity [40,69,73,74]. The development of novel CAIX inhibitors and clinical trials has been reviewed recently [8,28,29,30,75].

## 4. Other Possible Functions for CAIX in Cancer Progression

Although the main function of CAIX in cancer is as a regulator of pH_i_, several studies show that other possible mechanisms may be linked to this enzyme, thereby expanding its role in tumour progression. A recent study found novel roles for CAIX in tumour cell migration and MMP14-mediated invasion [67]. Interactions were identified with β1 integrins, metabolic transporters, integrin-associated protein CD98hc, and MMP14. CAIX appears to associate with MMP14 through phosphorylation sites in the intracellular domain of CAIX and can increase the degradation of collagen by MMP14 through providing the H^+^ the protease needs for its catalytic activity [67]. 

Interestingly, another recent report has linked MMP14 to the invasive capacity of breast cancer cell lines [66]. This study showed that although HIF-1α levels increased in hypoxic conditions, the expression of CAIX was variable between cell lines, and was only markedly upregulated in MCF-7 cells, a non-invasive cell line, after exposure to chronic hypoxia. HIF-1α gene expression can be constrained by FIH-1, which in turn can be inhibited by MMP14 [16]. Although MCF-7 cells expressed high FIH-1 levels, they lacked the active form of MMP14, suggesting that in this cell line, FIH-1 is able to prevent full HIF-1 activation in acute hypoxia, but not in chronic hypoxia [66]. This is in agreement with prior studies showing that FIH-1 suppression increases CAIX expression in hepatoma and osteosarcoma cell lines [14]. In the MCF-7 models, FIH-1 knockdown increased CAIX expression in hypoxic cells [66]. Taken together, these results suggest a strong link between hypoxia, HIF-1, FIH-1, CAIX, and MMP14 in the regulation of cancer cell invasive potential [14,16,66,67]. 

CAIX can decrease binding of E-cadherin to the cytoskeleton and affect cell adhesion [76] while also increasing the metastatic potential of tumour cells by effects on the activity of Rho-GTPase [77]. It similarly interacts with DKK1 protein, and thus the Rho/ROCK pathway, activating paxillin and stimulating migration [77]. CAIX can be phosphorylated at Thr-443 by Protein Kinase A during hypoxia, causing activation of CAIX and facilitating migration via increased transcription of proteins involved in cytoskeletal organisation and EMT [77,78,79]. Overexpression of CAIX also modulates Rho/ROCK signalling (which is pH sensitive), activates paxillin, increases focal adhesion turnover, cell migration, and activation of the FAK/PI3K/mTOR/p70S6K signalling pathway [77,80]. Conversely, CAIX inhibition impedes ROCK1 and decreases invasion [68,81]. 

## 5. Radiation

Radiotherapy is used to treat approximately 50% of cancer patients, either alone or in concert with chemotherapy or surgery [82]. It aims to eliminate cancer cells from the primary tumour, regional lymph nodes, or oligometastatic disease whilst limiting normal tissue damage. Radiation responses depend on the production of free radicals and intermediate ions that cause DNA damage in the form of single-strand (SSBs) or double-strand (DSBs) breaks in DNA. DBSs are the most effective in terms of inflicting cell damage and activate the DNA damage response (DDR) pathway that regulates whether the cell repairs DNA or undergoes cell death [83,84]. 

DSBs in DNA are detected by ataxia telangiectasia mutated (ATM) and ataxia telangiectasia and Rad3-related (ATR) kinases, which activate signalling pathways that stimulate cell cycle checkpoints and DNA repair. H2AX, once phosphorylated by ATM, recruits DNA repair proteins to the damaged area, and cyclins and cyclin-dependent kinases (CDKs) at G1/S and G2/M interphases delay cell division while DNA is repaired. The damage is restored by either homologous recombination or nonhomologous end joining (NHEJ) repair pathways. If DNA is not repaired, the damage results in cell death. 

However, some tumours may either acquire or possess intrinsic radioresistance, and a major clinical advantage would be achieved in cancer treatment if new approaches to sensitizing these tumours to radiotherapy were developed [84,85]. Studies in the 1950s acertained the role of hypoxia in radioresistance and, conversely, the role that O_2_ plays in radiation responses [86,87,88]. Maximal cell kill in response to radiotherapy needs O_2_ to form free radicals that damage DNA, and stabilise or ‘fix’ radiation-induced DNA damage [89]. This causes changes in the DNA that cannot be repaired, leading to cell death if the cell tries to undergo cell division [1,90]. Hypoxic cells can be 2 to 3 times more resistant to the same dose of radiation, because fewer DSBs are stabilised [88]. Another factor is the decrease in cell proliferation caused by hypoxia, as DNA damage is higher in rapidly dividing cells [5]. The phase of the cell cycle also affects radiation responses, with cells in G2/M and G1 phases the most radiosensitive and those in the S phase more radioresistant [91,92]. Increased acidification decreases the effectiveness of radiation; cells cultured in acidic media are more resistant to radiation, with acidic pH_e_ shown to reduce fixation of radiation-induced DNA damage, inhibit radiation-induced apoptosis, and delay G2/M-phase arrest allowing more time for treated cells to repair DNA damage, thus increasing radioresistance [7,93,94,95,96,97,98,99]. 

## 6. CAIX Inhibition and Radiation 

Studies have shown that CAIX can influence the response of cancers to radiation [53,100]. The knockdown of hypoxia-induced CAIX, or CAIX and CAXII together, sensitised tumour cells to radiation by decreasing the number of cells in the radioresistant S phase in both *in vitro* and *in vivo* models. This knockdown caused decreased intracellular pH values, which were found to correlate with enhanced cell death, suggesting that active CAIX is protecting cells against radiation by preserving an alkaline pH_i_, since ectopic CAIX expression increased cell survival after radiation treatment [29,62]. Recent studies have also shown the ability of CAIX inhibitors to sensitise renal cell carcinoma to radiation by increasing radiation-induced apoptosis [101], which is one mechanism known to be involved in the therapeutic effect of radiotherapy [102]. CAIX inhibitors similarly enhanced radiation sensitivity when used in combination in a breast cancer model; proteomic studies indicated that co-treatment increased expression of pro-apoptotic proteins and reduced expression of anti-apoptotic proteins [66]. Other pre-clinical data using xenograft models have shown that tumours expressing high levels of CAIX were less responsive to radiation, but that CAIX inhibitors could significantly increase radiosensitivity [103,104]. For example, the co-treatment of CAIX inhibitors with radiation in a colon HT29 mouse xenograft model demonstrated an improved therapeutic effect [103]. Although this was not apparent in *in vitro* studies, a novel class of sulfamate CAIX inhibitors enhanced the effects of radiation in a colorectal model, both *in vitro* and *in vivo* [103,104]. The use of isotopic substitution experiments could give insight into whether CAIX inhibition induces radiation sensitivity by increasing intracellular H^+^ concentrations [24].

## 7. CAIX and Radiation Responses, and Other Mechanisms

Although most research suggests that CAIX influences cancer responses via pH regulation, this enzyme can also interact with other mechanisms involved in cellular reactions to radiation, suggesting that additional factors may be involved in its ability to radiosensitize cancer cells. Radiation triggers several signalling cascades known to be involved in cell survival such as the PI3K/AKT and ERK pathways; this occurs through activation of the epidermal growth factor receptor (EGFR) [105,106]. EGFR can influence radiation responses by binding to DNA-dependent protein kinases (DNA-PK), enhancing their activity and thus DNA repair [106,107,108]. Radiation induces nuclear accumulation of EGFR, where it is involved in the relaxation of chromatin, allowing DNA repair proteins access and thus enhancing resistance to radiation [109]. Decreased expression of EGFR or AKT has been shown to increase radiation sensitivity in human cancer cells [110]. Activation of EGFR by epidermal growth factor causes phosphorylation of CAIX on Tyr449, which in turn can activate the PI3K/AKT pathway by interacting with the p85 regulatory subunit [111]. This suggests that radiation itself may activate survival pathways through a mechanism that at least partially involves CAIX. PI3K/Akt is one of the pathways stimulated by radiation that is known to be involved in the inhibition of cell death via apoptosis; further, several studies have also linked overexpression and activation of EGFR with radiation resistance in cancer [112,113,114,115,116,117,118]. EGFR inhibitors can sensitise cancer cells to radiation both *in vitro* and *in vivo,* with positive results also observed in a Phase III trial in head and neck cancer [119,120]. Whether this response to radiation is in part through the prevention of CAIX phosphorlyation and activation of PI3K/AKT is currently unclear. 

NF-κB activity is stimulated by hypoxia and acidic pH [121,122,123,124,125]. It is also activated by radiation and has a key role in radiation resistance and cell survival [126,127,128,129,130]. CAIX can interact with the NF-κB signalling pathway via a mechanism involving β1-integrin. Expression of β1 integrin is increased in various cancers, where it is involved in cell survival, proliferation, apoptosis, invasion, metastasis, and resistance to both chemotherapy and radiotherapy [131,132,133,134,135]. Studies have shown that cancer cells can be sensitized to radiotherapy by targeting β1 integrins [136,137]. The radioresistance mediated by β1 integrin is regulated by NF-κB, which increases β1-integrin expression, but conversely, inhibition of β1-integrin can inhibit the transcriptional activity of NF-κB [138]. A recent study demonstrated an interaction between CAIX and β1 integrin in tumour cells [67]; therefore this is another possible mode of interaction between CAIX and radiation responses. The downmodulation of β1 integrin can synergistically inhibit Akt-mediated survival in breast cancer cell lines and enhance radiotherapy in breast cancer xenografts [134,136,139]. A further study has shown that CAIX is required for the activation of NF-κB in hypoxia and can, via this interaction, stimulate the production of G-CSF to promote movement of granulocytic myeloid-derived suppressor cells (MDSC) to the metastatic niche of the lung [140]. Interestingly, G-CSF is strongly linked with protection from radiation damage [141,142], and high expression of the receptor is associated with poor response to radiotherapy in rectal cancer [143]. 

**Figure 3:**
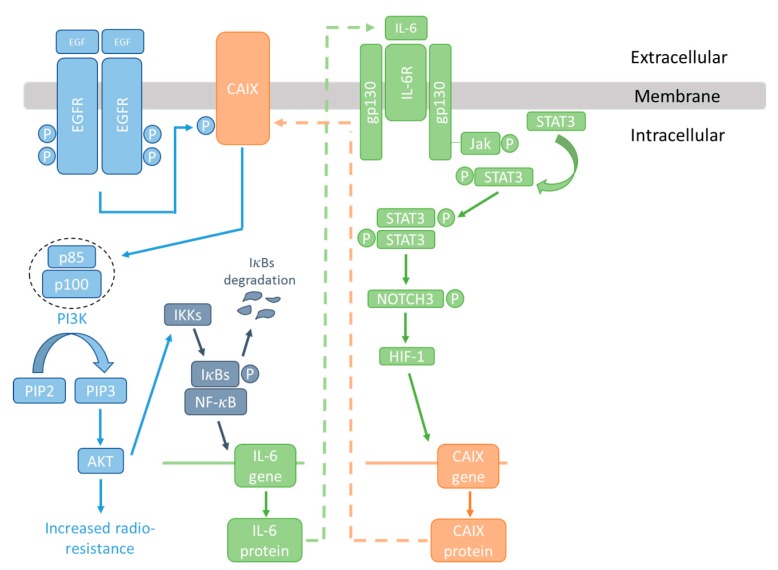
The CAIX/NF-κB/IL-6 signalling node and radioresistance.

Signal transducer and activator of transcription 3 (Stat3) is overexpressed in many cancers; STAT3 has been shown to be involved in the regulation of CAIX expression in several studies [144,145]. Inhibition of STAT3 has been found to increase radiation sensitivity in cancer cells, and to inhibit radiation-induced progression in glioma [146,147,148]. IL-6 promotes tumour growth and invasion through STAT3 activation [140], and has likewise been linked to radiation resistance [149,150]. IL-6 is also an NF-κB responsive gene [151]. Taken together, it is therefore possible that CAIX is part of an IL-6-STAT3-NF-κB signalling axis involved in radiation resistance as illustrated in Figure 3. These interactions appear to be independent of the pH-regulating roles of CAIX.

EGF induces phosphorylation of CAIX via the EGFR, allowing interaction with the p85 regulatory subunit of PI3K and activation of survival pathways. AKT can in turn activate I-κB kinase (IKK) and the NF-κB transcription factor, causing the production of IL-6. IL-6 can, via STAT3 and HIF, increase CAIX expression. Overexpression of CAIX, EGFR, STAT3, and IL-6, and activation of NF-κB, EGFR, and STAT3, have all been linked with radiation resistance.

The increased presence of lactate in the TME has been linked to both radiation and chemoresistance [36,152,153], which may be due to the antioxidant effects of lactate in the case of radioresistance [154]. The secretion of excess lactic acid from the cell is regulated by MCTs, such as the HIF1-inducible MCT4, which operates almost solely to export lactate, or MCT1, which also transports other monocarboxylates [18,155,156,157]. Both MCT1 and 4 co-transport H^+^ with lactate [18]. MCT1 expression is increased in various cancers such as ovarian, prostate, breast, and colorectal cancers, where it correlates with progression and poor patient prognosis [158,159,160]. In xenografts of lung, colorectal, or small cell lung cancer, MCT1 inhibitors decreased lactate secretion and increased radiosensitivity [161,162]. 

The MCT1/4 accessory molecule CD147 is required for plasma membrane expression of these transporters; if targeted, it can decrease expression of these MCTs and inhibit tumour growth in an *in vivo* model [163]; therefore, CD147 should also be linked to radiation responses. Studies show that CD147 can promote radioresistance in hepatocellular carcinoma cells *in vitro* and *in vivo*, and it has been linked to radioresistance in cervical cancer [164,165,166]. Interestingly, CD147 has also been demonstrated to interact with integrin β1 in hepatocellular carcinoma, causing activation of the FAK pathway and increasing malignancy of these cells.

Could CAIX inhibition affect lactate secretion from cancer cells and thus sensitise resistant tissues to radiation? In an interesting study, it was found that the increased lactate efflux from hypoxic breast cancer cells was not due to amplified expression of MCTs, but to a hypoxia-induced upregulation of CAIX, via a mechanism that was independent of its catalytic activity [37]. Other studies have also demonstrated that CAs have effects on cells that are not dependent on the catalytic activity of these enzymes. For example, it has been demonstrated that both the cytosolic CAII and CAIV can enhance the activity of monocarboxylate transporters 1 and 4 in a non-enzymatic manner, thus increasing lactate flux [34,36,167,168]. Jamali et al. showed that knock-down of CAIX decreased lactate flux by approximately 50%. This study proposed that CAIX may function as a ‘proton-collecting/distributing antenna’ that accelerates proton transfer and requires an extracellular location for CAIX, and that could facilitate both MCT1 and MCT4 activity. They further suggested that this collaboration between MCTs and CAIX would not be inhibited by compounds that specifically target CAIX catalytic activity [37].

Recently, lactate has been shown to induce the expression of CAIX in normoxic cancer cells both *in vitro* and *in vivo* in a mechanism involving both HIF-1 and specificity protein (SP-1) transcription factors [169]. The major mechanism of CAIX increase appeared to be through redox-dependent stabilisation of HIF-1α. Again, this suggests a possible signalling loop in which hypoxic cells produce lactate that can increase CAIX expression via HIF, and which in turn allows increased lactate export via CAIX/MCT co-operation and thus increased radiation resistance.

Hypoxia induces dedifferentiation of cancer cells to become phenotypically more stem cell-like [170]. It has been proposed that CAIX may be involved in this process, as CAIX expression has been shown to correlate well with that of CD44, a breast cancer stem cell marker [171,172,173]. Radioresistance is considered an inherent characteristic of cancer stem cells [174,175,176]. It has been suggested that such cells may repair DNA more effectively after radiation, since they express high levels of genes associated with DNA damage repair [177,178,179,180]. Inhibition of CAIX depletes the number of breast cancer stem cells in tumour hypoxic subvolumes, and therefore CAIX inhibitors may be useful to treat the radioresistant cancer stem cell population. Furthermore, it has been inferred that CAIX is required to maintain the stemness phenotype within the hypoxic niche of breast tumors [65]. This may be due to the possible mechanisms outlined above, or to the effect of CAIX on the acidic TME, since extracellular acidosis has also been linked to the development of ‘stemness’ [181]. Interestingly, increased lactate concentrations can also cause cancer cells to develop a cancer stem cell phenotype [182]. In pancreatic cancer stem cells, STAT3 is activated, but STAT3 inhibition decreases both radioresistance and stem cell numbers in pancreatic cancer [148]. Therefore, since both lactate and STAT3 activation can increase expression of CAIX [144,145,169], it is certainly possible that the effect of CAIX inhibitors on radiation sensitivity of cancer stem cells is due to the interactions of CAIX with lactate and STAT3.

## 8. Conclusions

CAIX is an attractive target for the treatment of cancer [8,31,32,33]. Data suggests that CAIX inhibition is a therapeutic strategy that could interfere with cancer cell proliferation, migration, and invasion, while *in vivo* studies demonstrate that metastatic growth could also be limited. While evidence indicates that the effectiveness of this inhibition is through interference with pH regulation in cancer cells, recent studies show that CAIX can interact with many other signalling pathways and mechanisms known to be active in cancer cells, many of which appear to influence the response of cancer cells to radiation. These pathways are not mutually exclusive, and sensitivity to radiation could be determined by additive or synergistic interactions between pH-dependent and independent mechanisms, which suggests that CAIX may have many important roles in cancer cells that could potentially be exploited therapeutically, particularly by radiation oncologists.
